# A Science Strategy for the Public Health Agency of Canada

**DOI:** 10.14745/ccdr.v51i05a01

**Published:** 2025-05-01

**Authors:** Sarah Viehbeck, Kimberly Girling, Erin Dunn

**Affiliations:** 1 Chief Science Officer and Vice President of Science and Policy Integration, Public Health Agency of Canada, Ottawa, ON

**Keywords:** public health, science strategy, emergency preparedness, data science, surveillance

A foundation of scientific excellence should be the base of a world-class public health system.

Science is at the core of the Public Health Agency of Canada’s (PHAC) mission to improve the health of all the people and communities within Canada. It is enshrined in our enabling legislation, which gives Canada’s Chief Public Health Officer the role of providing “the Minister and the President [of PHAC] with public health advice that is developed on a scientific basis”. As the national public health institution of Canada, the science we fund, conduct and use enables effective, evidence-informed decision-making, supports strong communication to the public and empowers people in Canada to take measures to protect and promote their health.

Science and research are essential to promote preparedness and resilience of public health systems. Through rigorous scientific activities, informed and contextualized through community engagement, we can understand public health threats and how they differ across and between populations, anticipate challenges, identify opportunities for equity-based solutions and respond effectively with tailored and relevant interventions. This work is often unseen, as the PHAC puts together early signals and acts in advance of emerging health threats to avert crises or minimize their impacts on people in Canada.

Scientific capacity, including a highly skilled science workforce supported by a series of robust enabling infrastructures, underpin Canada’s ability to anticipate and respond to emerging health threats and can serve as an insurance policy towards national preparedness, health security and innovation. While collaboration and coordination across the system in an emergency is imperative, it is crucial to have baseline capacity within the federal government to ensure it can rapidly respond to threats at the earliest moment of detection.

During the COVID-19 pandemic response, PHAC had to adapt our scientific activities to meet the pressing needs of a public health crisis. These adaptations included building up existing and strengthening new capacities, such as genomics, wastewater surveillance, behavioural sciences and serosurveillance, as well as fostering new approaches to link science to decision-making and public health guidance. These changes involved fostering new or existing science advisory tables and facilitating new approaches to communicate science. While Canada’s response to COVID-19 was strong, there is an opportunity to apply lessons learned and best practices from an acute emergency towards resilience and science excellence in our PHAC planning towards future public health challenges. Many of these lessons learned reports emphasized the importance of institutionalizing science advice systems, strengthening coordination and collaboration across jurisdictions and internal and external expertise, and improving readiness and efficiency, to be more resilient towards future challenges (1-3).

In December 2024, PHAC released its *PHAC Science Strategy 2024–2025 to 2029–2030: Advancing health, well-being, and equity through science* ((4)). Informed by robust domestic and international engagement, including dedicated dialogues with Canadian Indigenous communities, the Strategy reflects a purposeful shift towards a more open and collaborative model for our scientific work. Geared to be public-facing and with a global outlook, the Science Strategy aligns with other national public health science agendas and will support PHAC in the following areas:

• Planning and prioritizing scientific activities and guiding science investments

• Communicating clearly and transparently about science and how it is used in public health decisions and guidance

• Supporting the science workforce and capacity building

• Guiding scientific collaborations in a strategic and sustainable way

The Science Strategy takes a broad and inclusive view of science, including diverse disciplines from social and equity-based science to physical sciences and diverse ways of knowing, including Indigenous and cultural knowledge. It introduces a broadened definition of science at PHAC, developed through an antiracism lens, which embraces multiple ways of knowing, values integrity and fosters inclusion and partnership. This approach acknowledges that addressing complex public health challenges requires diverse perspectives and multidisciplinary solutions.

The Science Strategy articulates a clear value proposition for our science activities, positioning PHAC in the global public health landscape: leading and enabling science that is relevant, trustworthy and timely to policy and practice in the service of equitable public health impact at the national level. The science priorities it set focus on areas where PHAC is best positioned to contribute scientifically and can add the most value for people and communities in Canada:

• Advancing data science and the science of public health surveillance by innovating in surveillance methodologies and improving access to quality public health data

• Evolving the science of public health communication to better help people in Canada make informed decisions about their health based on strong, trusted scientific evidence

• Strengthening implementation science by studying how our public health programs and policies work to increase their impact

• Integrating science in public health emergencies by making sure science is at the heart of emergency preparedness, response and recovery

A cornerstone of this work is our commitment to meaningful collaboration with Indigenous partners. Through long-term reciprocal relationships, we aim to weave Indigenous knowledge with PHAC’s existing scientific practices, support the decolonization of public health science and take actionable steps to integrate anti-racism principles into our work.

This Science Strategy represents more than a set of priorities—it is a forward-looking vision of our science culture so that the science we lead and enable is transparent, collaborative and impactful. We cannot achieve this work in isolation; to advance our science priorities, we will look to create new and foster existing scientific partnerships and make our scientific activities more visible and operationally transparent. These strategic collaborations will support PHAC’s scientific work so that we can remain credible, timely, relevant and trustworthy into the future.
